# Relative Frequency and Distinctive Features of Anti-Ma2 Nonparaneoplastic Neurologic Disorders: A French Cohort Study

**DOI:** 10.1212/NXI.0000000000200538

**Published:** 2026-01-07

**Authors:** Mantas Vaisvilas, Gemma Lafuente-Gómez, Macarena Villagrán-García, Antonio Farina, Maxime Bonjour, Nicolás Lundahl Ciano-Petersen, Louis Comperat, Geraldine Picard, Dimitri Psimaras, Bastien Joubert, Jerome Honnorat

**Affiliations:** 1 French Reference Center on Paraneoplastic Neurological Syndromes and Autoimmune Encephalitis, Hospices Civils de Lyon, Hôpital Neurologique, Bron, France;; 2 SynatAc Team, Institute MeLiS INSERM U1314/CNRS UMR 5284, Université de Lyon, Université Claude Bernard Lyon 1, France;; 3 Clinic of Neurology and Neurosurgery, Institute of Clinical Medicine, Faculty of Medicine, Vilnius University, Lithuania;; 4 Department of Biostatistics, Hospices Civils de Lyon, Laboratoire de Biométrie et Biologie Évolutive, UMR CNRS 5558, University Claude Bernard Lyon 1, Villeurbanne, France;; 5 Neuroimmunology and Neuroinflammation Group. Institute of Biomedical Research of Málaga - IBIMA, Málaga, Spain;; 6 Red Andaluza de Investigación Clínica y Traslacional en Neurología (NeuroRECA), Málaga, Spain;; 7 AP-HP, Hospital Group Pitié-Salpêtrière, Neuro-oncology Department Paris, France; and; 8 Inserm U1127, CNRS, Paris Brain Institute, Institut du Cerveau (ICM), France.

## Abstract

**Background and Objectives:**

Although anti-Ma2 antibodies (Ma2-Abs) typically associate with paraneoplastic encephalitis, a subset of patients with Ma2-Abs does not have any detectable tumor. The clinical specificities of these idiopathic cases are unknown. The aim of this study was to describe clinical phenotypes and outcomes of patients with idiopathic Ma2-Abs (I-Ma2) compared with patients with paraneoplastic Ma2-Abs (PNS-Ma2).

**Methods:**

A retrospective review of the French Reference Center of Paraneoplastic Neurologic Syndromes and Autoimmune Encephalitis database was conducted to identify cases of Ma2-Abs–mediated syndromes diagnosed between January 2002 and December 2022. A systematic review of the existing literature was also performed to assess for reported I-Ma2 cases.

**Results:**

Seventy patients with neurologic syndromes harboring Ma2-Abs were identified (50/70 men, 71.4%; median age 60 years, interquartile range (IQR) 47–67.5). Malignancies were detected in 46 of 70 (65.7%). When compared with the PNS-Ma2 cohort, the I-Ma2 cohort less frequently had an acute/subacute disease progression (5/20, 25%, vs 26/46, 56.5%; *p* = 0.037) and the time to diagnosis was longer (10 months, IQR 4–20, vs 3.5 months, IQR 1–6; *p* = 7.94 x 10^−10^). No differences were found in the subtype of clinical symptoms between patients with and without cancer. However, monofocal involvement, predominantly isolated limbic encephalitis, was more frequent in patients with I-Ma2 vs the PNS-Ma2 cohort (13/20, 65%, vs 18/46, 39.1%; *p* = 0.05). The other discriminative paraclinical finding was EEG alteration, which was more frequently abnormal in patients with I-Ma2 (I-Ma2 11/20, 55%, vs PNS-Ma2 12/46, 26%; *p* = 0.047). There were no differences in brain MRI and CSF inflammation. Systematic review of the literature revealed a longer follow-up, a greater use of second-line immunotherapies, and a higher proportion of patients with I-Ma2 in the French cohort (proportion of French I-Ma2 20/66 (30.3%) vs 10/109 (10.9%) in the literature, *p* = 0.00325, respectively).

**Discussion:**

Patients with I-Ma2 are more common than anticipated and have insidious disease onset and a tendency for monofocal nervous system involvement. Recognition of patients with I-Ma2 is delayed and must be improved.

## Introduction

Anti-Ma2 antibodies (Ma2-Abs) typically associate with a clinical syndrome of paraneoplastic limbic encephalitis, isolated or combined with cerebellar, brainstem, or diencephalic involvement, and have been described mostly in male individuals with testicular or lung malignancies.^1-3^ However, over the past 2 decades, other clinical presentations, including narcolepsy, movement disorders, motor neuron disease–like presentations, myelopathy, and polyneuropathy,^4-9^ as well as different types of cancers, including hematologic, gynecologic, urologic, esophageal, or tonsillar tumors, have been reported in patients with Ma2-Abs.^4,10-15^ Remarkably, there is also a subset of patients who do not have any detectable cancer and were reported under the term of idiopathic Ma2 autoimmunity.^13^ Data from such cancer-free patients are limited to case reports with inconsistent reporting of follow-up and outcomes.^6,13,16-18^ To date, the relative frequency of such idiopathic presentations of Ma2 autoimmunity and whether patients with idiopathic Ma2-Abs (I-Ma2) differ from patients with paraneoplastic Ma2-Abs (PNS-Ma2) regarding clinical presentation, severity, or outcome remain unclear. In this study, we report the clinical features of a series of previously unpublished patients harboring Ma2-Abs, with the aim of comparing I-Ma2 and PNS-Ma2. We systematically reviewed the existing literature and compared the French cohort with previously published patients to assess for clinico-epidemiologic differences between patients with I-Ma2 and those with PNS-Ma2.

## Methods

### Patients

We retrospectively screened the database of the French Reference Center of Paraneoplastic Neurologic Syndromes and Autoimmune Encephalitis (Lyon, France), searching for patients harboring Ma2-Abs in the serum and/or CSF between January 2002 and December 2022. Samples were considered positive if they stained the nucleus and cytoplasm of neurons on indirect immunofluorescence of rat brain sections and were positive on a confirmation test (in-house cell-based binding assay, commercial immunodot, or Western blotting with recombinant Ma2 protein), as reported elsewhere.^19,20^ Available biological samples were further retrospectively tested for Ma1 antibodies using a commercial dot blot and in-house cell-based assay. Patients tested positive for Ma2-Abs in the context of immune checkpoint inhibitor treatment were previously reported and not included in the analysis.^21^ Clinical syndromes were classified according to the updated paraneoplastic neurologic syndrome criteria^22^ and based on neuraxial deficit (cerebellar syndrome, diencephalic syndrome, etc). Clinical and follow-up data were extracted from the medical charts of the patients. Acute cases were classified as those reaching symptom nadir within a month of onset, subacute cases within 3 months of onset, and chronic cases as taking more than 3 months to reach nadir. Modified Rankin Scale (mRS) scores were assessed retrospectively using medical charts. Stabilization of disease after immunotherapy was defined as no change in mRS scores 12 months after immunotherapy, whereas improvement was defined as decrease in mRS scores by at least 1 point 12 months after immunotherapy compared with baseline. Patients with insufficient clinical and follow-up data were excluded from the analysis. Patients with I-Ma2 were defined as positive for Ma2 antibodies but negative for malignancy after periodic screening, including regular full-body CT/PET-CT scans, with a follow-up of at least 2 years after diagnosis.

### Literature Search

A systematic literature review of patients with Ma2-Abs published between 1999 and 2022 was performed to compare demographic, clinical, and follow-up data from the literature with those of the French cohort. A flowchart is shown in eAppendix 1. Only patients harboring Ma2 antibodies but without evidence of cancer were included in the analysis. For patient selection, the following criteria were applied: Ma2 antibodies detected in brain tissue sections, followed by confirmatory assays and no evidence of cancer on initial screening via whole-body CT or PET-CT, and a minimum follow-up period of 12 months during which additional screening tests were performed.

### Statistical Analysis

Statistical analysis was performed using IBM SPSS Statistics for Windows, Version 26 (IBM SPSS Statistics for Windows, IBM Corporation, Armonk, NY). Qualitative variables were expressed as absolute frequencies and percentages. Continuous data were reported as mean and SD or as median and interquartile range (IQR), as appropriate. The Student *t* test or Mann-Whitney *U* test was used for comparing continuous variables, as appropriate, and χ^2^ test for categorical variables. *p* < 0.05 was considered statistically significant. UpSet plots were calibrated using the UpSetR package (version 1.4.0) in R (version 4.1.2).

### Ethical Considerations

All patients provided written informed consent for the storage and use of their serum, CSF, and clinical information for research purposes. The study was approved by the Institutional Review Board of the University Claude Bernard Lyon 1 and Hospices Civils de Lyon (ICARE NCT-03963700).

### Data Availability

All anonymized data are available on reasonable request to the corresponding author.

## Results

### Description of the Entire Cohort

Seventy patients with neurologic syndromes harboring Ma2-Abs were retrospectively identified (50/70 men, 71.4%; median age 60 years, IQR 47–67.5). For patients with detectable cancer, neurologic symptoms antedated cancer diagnosis in 28 of 46 cases (60.9%). A progressive disease course (symptoms evolving for >3 months) was observed in 46 of 70 patients (65.71%), with a median symptom progression of 4 months (IQR 1–24) to reach a fully established clinical syndrome. Monofocal involvement of a single nervous system region was seen in 31 of 70 patients (44.2%). Limbic system impairments predominated (47/70, 67.1%), and monofocal limbic impairment was observed in only 19 of 47 cases (40.4%).

**Figure 1:**
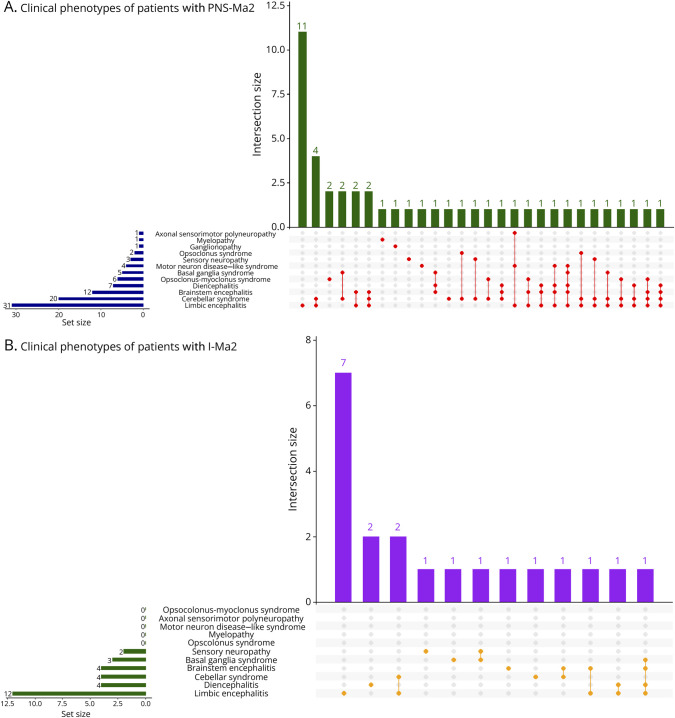
UpSet Plot for I-Ma2 and PNS-Ma2 Showing Clinical Phenotypes Patients with PrPNS-Ma2 are not included in the image. (A) Clinical phenotypes of patients with PNS-Ma2. (B) Clinical phenotypes of patients with I-Ma2. PNS-Ma2 = paraneoplastic Ma2-Abs; I-Ma2 = idiopathic (nonparaneoplastic) Ma2-Abs.

Other clinical manifestations (Figure 1) included movement disorders (6/70, 8.6%), with bradykinesia and rigidity (5/6, 83.3%) or chorea (1/6, 16.7%). Peripheral neuropathy was found in 8 of 70 cases (11.4%), characterized by motor weakness (3/8, 37.5%), sensory loss (5/8, 62.5%), proprioceptive ataxia (1/8, 12.5%), and/or amyotrophy (3/8, 37.5%).

In total, brain MRI was performed in 69 of 70 patients. Detailed information was available for 64 of 69 patients (92.8%). Disease-related abnormalities were present in 47 of 69 patients (68.1%). Detailed brain MRI characteristics of the entire cohort are presented in Table 1. CSF analyses were available for 63 of 70 patients (90%) and were abnormal in 47 of 63 (74.6%). Inflammatory CSF (pleocytosis and/or oligoclonal bands) was observed in 46 of 63 patients (73.1%) (Table 2).

**Table 1:** Description of MRI Abnormalities

Total number of encephalitis-related MRI abnormalities, N (%) 47/64 (73.4)						
Location of MRI abnormality	Hippocampus	Mesencephalon	Diencephalon	Cerebellum	Other nervous system areas	Multifocal involvement
Total abnormal, N (%)	30/47 (63.8)	6/47(12.7)	8/47 (17.1)	2/47(4.3)	1/47 (2.2)	7/47 (14.9)
FLAIR hyperintensity, N (%)	Unilateral 9/30 (30) Bilateral 21/30 (70)	5/6 (83.3) Cerebellar peduncles 2/5(40) Tegmentum 3/5 (50)	Thalamus 7/8 (87.5) Hypothalamus 1/8 (12.5)	0/2 (0)	Epiconus/conus lesion 1/45 (2.2)	—
Contrast enhancement, N (%)	7/30 (23.3)	0/5 (0)	1/8 (12.5)	0/2 (0)	1/45 (2.2)	1/7 (14.2)
Pattern of contrast enhancement, N (%)	Focal parenchymal 6/7 (85.7) Diffuse pachymeningeal 1/7 (14.3)	—	Focal parenchymal hypothalamic enhancement	—	Focal parenchymal conus/epiconus spinal cord enhancement	Concomitant focal parenchymal enhancement of the thalamus and mesial temporal lobe
Other relevant findings, N (%)	Hippocampal atrophy without T2-FLAIR hyperintensity, diffusion restriction, or contrast enhancement 3/30 (10) Bilateral atrophy 2/3 (66.7) Unilateral atrophy 1/3 (33.3)	Atrophy without T2-FLAIR hyperintensity, diffusion restriction, or contrast enhancement 1/6 (16.6)	—	Severe cerebellar hemispheric and/or vermian atrophy, one each	—	T2 FLAIR hyperintensity in mesio-temporal lobe structures and mesencephalic structures in 5/7 (71.4) T2 FLAIR hypersignal in mesio-temporal lobe structures and diencephalic structures in 2/7 (28.6)

Detailed information of abnormal MRI patterns was available in 64 of 70 cases (91.4%).

**Table 2:** Clinico-Demographic Data of the Cohort

	Ma2 without cancer N 20	Ma2 with cancer N 46	*p* Value
Male sex, n (%)	13 (65)	35 (76.09)	0.23
Age, median (IQR)	59 (49–64.25)	61.5 (47–67.5)	0.665
Neurologic symptoms at presentation, n (%)			
Cognitive decline	14 (70)	28 (60.9)	0.667
Seizures	7 (35)	8 (17.4)	0.826
Psychiatric symptoms	6 (30)	21 (45.7)	0.360
Diencephalic symptoms	6 (30)	6 (13)	0.162
Oculomotor palsy	6 (30)	25 (54.3)	0.120
Cerebellar syndrome	4 (20)	20 (43.5)	0.123
Sensitive symptoms	2 (10.5)	7 (15.2)	1.000
Dysautonomia	2 (10.5)	2 (4.3)	0.579
Endocrinologic alterations	2 (10)	5 (10.9)	1.000
Peripheral neuropathy	3 (15)	5 (10.9)	0.216
Disease onset, n (%)			
Acute/subacute (symptoms for 1–3 mo)	5 (25)	26 (56.52)	0.037
Progressive (symptoms for >3 mo)	13 (65)	18 (39.13)	0.096
Not specified	2 (10)	2 (4.35)	0.579
Time from onset to diagnosis in months, median (IQR)	10 (4–20)	3.5 (1–6)	7.9x10^−^^10^
Abnormal MRI, n (%)	13 (65)	34 (73.9)	0.416
Additional neural antibodies, n (%)	2 (10)	12 (26.1)	0.197
CSF changes			
Normal CSF^a^	4 (20)	10 (21.7)	0.832
White blood cell count >5/mm^3^, n (%)	10 (50)	23 (50)	1
Protein level >60 g/dL, n (%)	10 (50)	25 (54.35)	0.955
Oligoclonal bands, n (%)	7 (35)	22 (47.82)	0.487
Abnormal EEG, n (%)	11 (55)	12 (26.09)	0.047
Coexisting antibodies	2 (10)	14 (30.4)	0.141
Ma1	1 (50)	7 (50)	
Other	1 (50)^b^	7(50)^c^	
Treatment			
First-line immunotherapy, n (%)^d^	15 (75)	39 (84.8)	0.488
Second-line immunotherapy n, (%)	11 (55)	22 (47.83)	0.789
Rituximab, n (%)	7/11 (63.7)	10/22 (45.5)	0.324
Cyclophosphamide, n (%)	11/11 (100)	19/22 (86.3)	0.593
Steroid-sparing agents n, (%)^e^	1 (12.5)	1 (2.9)	1.0
Functional status			
mRS score 0–2 at presentation, n (%)	6 (30)	16 (34.78)	0.782
Median mRS score at presentation, (IQR)	3 (2–4)	3 (2–4)	0.591
Median mRS score at 12-mo follow-up, n (IQR)	3 (2–4.5)	3.5 (2–6)	0.682
Median mRS score at last follow-up	1 (1–2)	2 (1–3)	0.739
Mortality at 5-y follow-up, n/N (%)	9/19 (47.4)	26/40 (65)	0.260
Median follow-up, months (IQR)	103.58 (49.81–141.2)	66.25 (11.33–111.45)	5.6x10-7

Abbreviations: EEG = electroencephalography; mRS = modified Rankin Scale.

a No pleocytosis and/or absence of oligoclonal bands.

b One anti-Ri.

c One each (9.8%) against Yo, Zic4, voltage-gated calcium channel, LGI-1, and KLHL-11 and 2 of 14 (18.8%) against unidentified neuronal antigens.

d Includes high-dose methylprednisolone, IVIG, and plasma exchange alone or in combination.

e Mycophenolate mofetil or azathioprine.

### Cancer Identification and Case Definitions

After initial cancer screening, malignancies were detected in 46 of 70 patients (74.6%). The median delay of cancer diagnosis was 3 months (IQR 1–8). Patients with evidence of cancer on screening at any study time point were termed paraneoplastic Ma2 (PNS-Ma2) cases. In our series, 13 of 35 men with PNS-Ma2 (37.4%) were younger than 50 years and 12 of these 13 (92.3%) had testicular tumors. Detailed cancer descriptions, relative frequencies for men and women, and specific cancer therapies are given in Table 3. Relative frequencies according to antibody status and sex are provided in eTable 1.

**Table 3:** Distribution of Cancers With Ma2 Autoimmunity

Cancer type	Frequency, N (%) N = 46	Frequency in men, N (%) N = 35	Frequency in women, N (%) N = 11	*p* Value
Lung	15 (32.6)	10 (28.6)	5 (45.4)	0.456
Adenocarcinoma	8 (53.3)	4 (40)	4 (80)	
Squamous cell carcinoma	3 (20)	2 (20)	1 (20)	
Small-cell lung cancer	4 (26.7)	3 (30)	0	
Testicular	13 (28.3)	13 (37.1)	0 (0)	
Nonseminoma	7 (53.8)	7 (53.8)		
Seminoma	4 (30.8)	4 (30.8)		
Mixed germ cell tumor	2 (15.4)	2 (15.4)		
Mediastinal seminomatous germ cell tumor	1 (7.7)	1 (7.7)		
Head and neck cancers	3 (6.5)	3 (8.8)	0 (0)	1
Undifferentiated nasopharyngeal cancer	1 (33.3)	1 (33.3)		
Ethmoidal carcinoma	1 (33.3)	1 (33.3)		
Squamous cell tonsillar carcinoma	1 (33.3)	1 (33.3)		
Hematologic malignancies	2 (4.3)	1 (2.9)	1 (9.1)	0.425
Non-Hodgkin lymphoma	1 (50)	1 (100)	0 (0)	
Hodgkin lymphoma	1 (50)	0 (0)	1 (100)	
Skin malignancies	1 (2.2)	0 (0)	1 (9.1)	0.239
Melanoma	1 (100)		1 (100)	
Pleural mesothelioma	1 (2.2)	1 (0.03)	0 (0)	1
Digestive tract	7 (15.2)	6 (17.1)	1 (9.1)	1
Gastric adenocarcinoma	2 (42.9)	2 (33.3)	0 (0)	
Hepatic adenocarcinoma	2 (28.6)	1 (16.7)	1 (100)	
Papillar adenocarcinoma	1 (14.3)	1 (16.7)	0 (0)	
Esophageal carcinoma	2 (28.6)	2 (33.3)	0(0)	
Urologic malignancies	1 (2.2)	0 (0)	1 (9.1)	0.239
Renal carcinoma	1 (100)		1 (100)	
Gynecologic malignancies	2 (4.3)	0 (0)	2 (18.2)	
Ovarian carcinoma	1 (50)		1 (50)	
Breast cancer (adenocarcinoma)	1 (50)		1 (50)	
Unknown	1 (2.2)	1 (2.9)	0 (0)	1
Cancer therapies, N (%)	45/46 (97.8)			
Chemotherapy, n/N (%)	31/46 (67.4)			
Surgery, n/N (%)	24/45 (53.3)			
Radiation therapy n/N (%)	14/45 (31.1)			
Not specified, N (%)	3/45 (6.7)			

**Figure 2:**
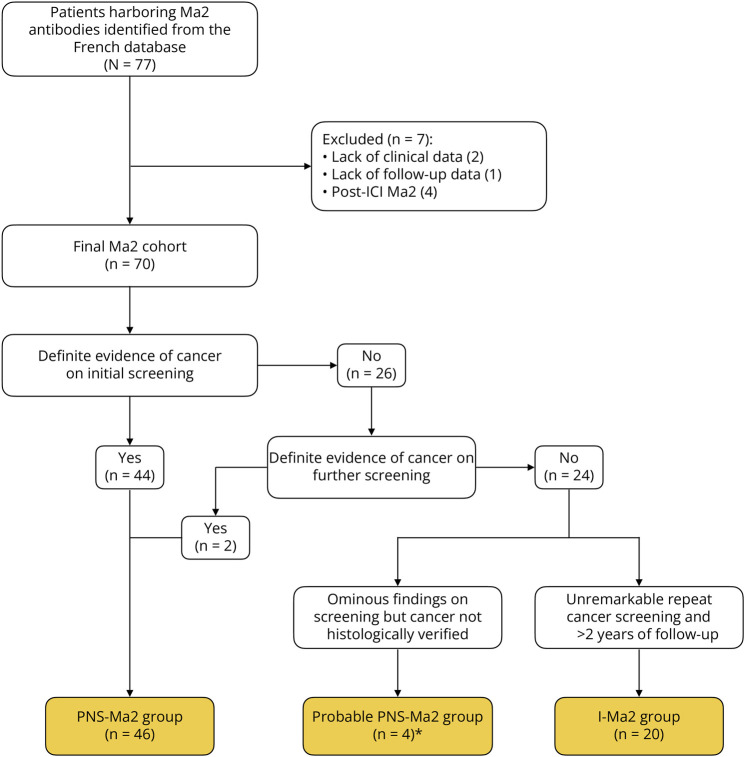
Selection of the French Ma2 Cohort I-Ma2 = idiopathic Ma2-Abs; PrPNS-Ma2 = probable paraneoplastic Ma2-Abs; PNS-Ma2 = paraneoplastic Ma2-Abs. *PrPNS-Ma2 cases not included in the analysis.

By contrast, 20 of 70 patients (28.6%) had no evidence of cancer on repeated screening at any time point and had a minimum follow-up of 2 years after diagnosis of Ma2 syndrome. Among them, 12 men with suspected testicular cancer had a median of 4 (IQR 2–6) testicular ultrasounds over the 2 years after diagnosis. Testicular microcalcifications were present in only one patient with otherwise negative cancer screening. Orchiectomy was proposed, but the patient refused. In another patient, bilateral orchiectomy was performed without evidence of cancer on histology. Women were examined using gynecologic ultrasound and mammography, with a median of 3 (IQR 1–6) examinations over 2 years. Examinations beyond testicular and gynecologic cancers included full-body CT in 11 of 20 (55%) or PET-CT scans in 9 of 20 (45%), with a median of 5 (IQR 2–7) tests over 2 years after diagnosis, and all results were negative. A flowchart depicting patient selection is shown in Figure 2.

### Comparison Between PNS-Ma2 and I-Ma2 Cohorts

Patients with I-Ma2 had a chronic course of disease compared with those with PNS-Ma2 (15/20, 75%, vs 20/46, 43.5%; *p* = 0.037), and the time to diagnosis from the onset of symptoms was longer in the I-Ma2 cohort (median 10 months, IQR 5–20) than in the PNS-Ma2 cohort (3.5 months, IQR 1–6; *p* = 5.58 × 10^−10^). Monofocal involvement, predominantly isolated limbic encephalitis, was more frequent in patients with I-Ma2 vs those with PNS-Ma2 (13/20, 65%, vs 18/46, 39.1%; *p* = 0.05). The only discriminative paraclinical finding was EEG alteration, which was more frequently abnormal in patients with I-Ma2 (I-Ma2 11/20, 55%, vs PNS-Ma2 12/46, 26.1%; *p* = 0.047).

**Figure 3:**
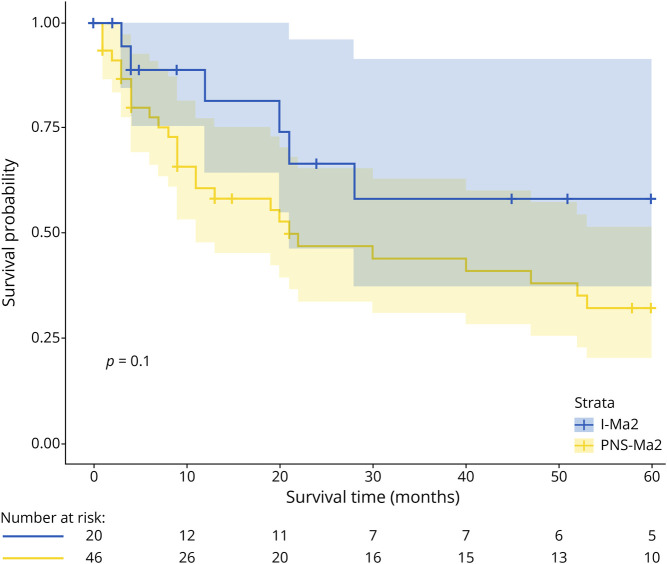
Kaplan-Meier Curve for PNS-Ma2 and I-Ma2 Cohorts

Similar to time to diagnosis, the time to lumbar puncture was longer in the I-Ma2 cohort compared with the PNS-Ma2 cohort, but the proportion of patients with normal CSF did not differ (Table 2). A similar proportion of patients with I-Ma2 and PNS-Ma2 received first-line and second-line immunotherapies (15/20 (75%) vs 39/46 (84.7%), *p* = 0.488; 11/20 (55%) vs 22/46 (47.8%), *p* = 0.789, respectively). Effectiveness of immunotherapies measured by functional independence (defined as mRS scores 0–≤2) at last-follow up did not differ between the 2 groups (7/11 (63.7%) for I-Ma2 vs 11/14 (78.6%) for PNS-Ma2, *p* = 0.656, respectively). Survival analysis revealed that of 26 recorded deaths in the PNS-Ma2 group, 16 (61.5%) of these patients died within the first 12 months after the onset of symptoms. By contrast, only 2 of 9 patients (22.2%) in the I-Ma2 group died during the same period (*p* > 0.05). There was a tendency toward better overall survival at 60 months for patients with I-Ma2 compared with the PNS-Ma2 group (58.3% vs 32.3%, *p* = 0.1, Figure 3). In one patient of the I-Ma2 group, autopsy revealed findings suggestive of isolated brain sarcoidosis without evidence of cancer.^23^

### Concomitant Ma1 and Other Autoantibodies

In total, 42 of 70 patients (60%) were tested for concomitant Ma1 antibodies, and only 7 of 42 (16.6%) were positive (eTable 2). Together, 16 of 66 patients (24.2%) had concomitant neural antibodies (Table 2).

### Review of the Literature

A systematic review was conducted to compare patients with I-Ma2 reported in the literature with the French cohort (Table 4). We observed a longer follow-up, a greater use of second-line immunotherapies, and a higher proportion of patients with I-Ma2 in the French cohort (proportion of French I-Ma2, 20/66 (30.3%) vs 10/109 (10.9%) in the literature, *p* = 0.00325, respectively). Subgroup analysis comparing patients with only Ma2 antibodies showed a higher incidence of nontesticular tumors and a higher number of I-Ma2 cases in the French cohort (eTable 3).

**Table 4:** Comparison of the Present Series With Patients Reported in the Literature

	I-Ma2 French cohort N= 20	I-Ma2 literature cohort N = 10	*p* Value
Age, median (IQR)	59 (45–69)	60 (45–68)	1.00
Monofocal nervous system involvement, N (%)	13/20 (65)	6/10 (60)	0.431
Any limbic involvement, N (%)	11/20 (55)	4/10 (40)	0.700
Proportion treated with immunotherapy, N (%)	15/19 (78.9)	7/8 (87.5)	1.00
First-line immunotherapy, N (%)	15/15 (100)	7/8 (87.5)	0.296
Second-line immunotherapy, N (%)	11/15 (73.3)	2/8 (25)	0.04
Median follow-up, months (IQR)	103.58 (49.81–141.2)	24 (12–42)	0.00019
Proportion deceased at last follow-up, N (%)	9/19 (47.3)	3/10 (30)	0.449

## Discussion

This study describes a nationwide retrospective cohort of patients with Ma2-Abs and compares clinical findings depending on the presence of detectable cancer. In concordance with previous studies, a paraneoplastic etiology was established in most of the cohort.^1,2,22^ However, we identified a substantial proportion of patients without identifiable cancer after 2 years of follow-up, supporting prior findings of isolated reports that suggested the existence of an ‘idiopathic’ form of Ma2-Abs autoimmunity.^13,16,17,24^

First, the present data confirm that cases of Ma2-Abs autoimmunity can occur without any detectable cancer (i.e., patients with I-Ma2) and are, in fact, more common than previously anticipated, comprising nearly a third of patients with Ma2-Abs. This starkly contrasts with other antibodies considered at high risk of an underlying malignancy (i.e., Yo, Hu, and Ri), in which the proportion of patients without any detectable tumor is consistently around 10%.^25-27^

For patients with confirmed malignancies, testicular seminoma was initially identified as the tumor most commonly associated with anti-Ma2 antibodies.^1^ However, our study indicates an expansion in the repertoire of tumors beyond the testis, with testicular seminoma present in only one-third of men, regardless of isolated Ma2 or combined Ma1+Ma2 positivity (eTable 3). By contrast, lung carcinoma was the most frequently observed associated cancer among women and was also prevalent in men. In addition, we observed unexpectedly high variability in tumor types within our cohort. Approximately half of the patients had somatic and hematologic malignancies that were neither lung nor testicular cancers. This finding contrasts with earlier reports, which indicated that lung and testicular malignancies were observed in two-thirds of cases.^2^ However, tumor associations in Ma2-mediated neurologic syndromes heavily rely on the co-occurrence of antibodies to concomitant Ma2 isoforms. Earlier studies report the frequency of co-occurrence of either Ma1 or Ma3 positivity to be as high as 40%.^2,13,28^ This contrasts to our study because only 17% of the cohort had co-occurring Ma1 antibodies. This finding is likely due to the lack of systematic testing for Ma1 antibodies and may have limited our capacity to observe true relative frequencies of Ma1/2 antibodies and their cancer associations. Although we report various clinical phenotypes and an extended repertoire of tumors similar to historic cohorts describing patients with coexisting Ma2 and Ma1 antibodies,^1,29^ we cannot confirm that this observation is solely due to Ma1 antibodies, because we did not systematically screen each patient for the presence of Ma1. Consequently, systematic testing for Ma1/Ma3 in specialized laboratories may further advance cancer screening and prevent misdiagnosis because recent studies suggest that both sensitivity and specificity of commercial assays to detect Ma2 antibodies are low.^20,30,31^ Nevertheless, regardless of Ma1/2 positivity, cancer screening should not be limited to the pulmonary and urogenital systems. Systematic testing for Ma1 antibodies, alongside PET-CT scans, may be warranted to facilitate diagnosis. It is important to note that negative scans should prompt consideration of orchiectomy in men younger than 50 years because intratubular germinal neoplasm is likely in the presence of isolated Ma2 antibodies.^32^

Second, we observed that patients with I-Ma2 more frequently present with an insidious and chronic disease course, as well as a tendency for monofocal CNS involvement. Similarly, the time to diagnosis is often longer for patients without cancer (I-Ma2), which suggests that chronically ill patients may not undergo testing for Ma2 autoimmunity. Clinicians should be aware that both I-Ma2 and PNS-Ma2 autoimmunity can occur in patients who experience an insidious onset of neurologic symptoms. Attention to these cases is crucial to ensure timely antibody testing and immunotherapy, which could result in optimal outcomes.

Notably, the demographic and clinical specificities of patients with I-Ma2 allow us speculate that the immunologic mechanisms driving idiopathic Ma2 autoimmunity might not be equivalent to those occurring in the paraneoplastic context. In a number of I-Ma2 cases from the French cohort, as well as in cases reported in the literature, associations with inflammatory conditions and connective tissue disease (Sjogren syndrome) have been reported.^16,23,33^ These findings support the idea that various immunologic mechanisms beyond paraneoplastic anti-tumor immune response could be involved in the formation of I-Ma2.

This study has some limitations, including a relatively small sample size of idiopathic cases, a retrospective methodology, and the challenges associated with the diagnostic criteria for paraneoplastic neurologic syndromes. Although most cancers are diagnosed within 2 years of the onset of these syndromes, it is still possible to identify malignancies beyond this time frame. This raises questions about the validity of classifying certain cases as idiopathic in the context of high-risk antibody-associated neurologic syndromes. Owing to the unavailability of samples, we were only able to test 60% of our patients for Ma1 antibodies. This might have limited our capacity to observe differences in cancer associations according to Ma1/Ma2 status and influenced the notably low number of Ma1-positive cases compared with historical cohorts.^1,2,13,28^ Despite these limitations, the main finding of this article is that the existence and increased frequency of I-Ma2 cases remain unaffected by the unavailability of samples and Ma1 serostatus.

Among patients with Ma2 antibodies, testicular seminoma occurs in approximately one-third of men while lung cancer is often seen in women. Approximately half of the patients may have somatic and hematologic malignancies unrelated to lung or testicular cancers, while patients with I-Ma2 show no detectable malignancies even after thorough follow-up. I-Ma2 typically presents with gradual onset and monofocal nervous system involvement, suggesting a different immunologic response compared with PNS-Ma2 cases. Although the treatment window may be extended for patients with I-Ma2, the lack of response to aggressive therapies at diagnosis underscores the need for early identification. These findings highlight the importance of consistent reporting of I-Ma2 cases in the literature to enhance understanding and facilitate earlier diagnosis and treatment for better patient outcomes.

## Glossary

IQR: interquartile range
Ma2-Abs: anti-Ma2 antibodies
mRS: modified Rankin Scale
